# Novel Binding Partners for CCT and PhLP1 Suggest a Common Folding Mechanism for WD40 Proteins with a 7-Bladed Beta-Propeller Structure

**DOI:** 10.3390/proteomes9040040

**Published:** 2021-10-02

**Authors:** Wai Shun Mak, Tsz Ming Tsang, Tsz Yin Chan, Georgi L. Lukov

**Affiliations:** 1 Faculty of Sciences, Brigham Young University-Hawaii, Laie, HI 96762, USA; wilsonctr@yahoo.com.hk (W.S.M.); jeremytmtsang@outlook.com (T.M.T.); tszyinchan@outlook.com (T.Y.C.); 2 Genome Center, University of California Davis, Davis, CA 95616, USA; 3 Department of Chemistry and Biochemistry, Brigham Young University, Provo, UT 84602, USA

**Keywords:** WD40, β-propeller, CCT, PhLP1, protein folding, protein motif, protein interactions

## Abstract

This study investigates whether selected WD40 proteins with a 7-bladed β-propeller structure, similar to that of the β subunit of the G protein heterotrimer, interact with the cytosolic chaperonin CCT and its known binding partner, PhLP1. Previous studies have shown that CCT is required for the folding of the Gβ subunit and other WD40 proteins. The role of PhLP1 in the folding of Gβ has also been established, but it is unknown if PhLP1 assists in the folding of other Gβ-like proteins. The binding of three Gβ-like proteins, TBL2, MLST8 and CDC20, to CCT and PhLP1, was demonstrated in this study. Co-immunoprecipitation assays identified one novel binding partner for CCT and three new interactors for PhLP1. All three of the studied proteins interact with CCT and PhLP1, suggesting that these proteins may have a folding machinery in common with that of Gβ and that the well-established Gβ folding mechanism may have significantly broader biological implications than previously thought. These findings contribute to continuous efforts to determine common traits and unique differences in the folding mechanism of the WD40 β-propeller protein family, and the role PhLP1 has in this process.

## 1. Introduction

The cytosolic chaperonin containing tailless complex polypeptide 1 (TCP-1), or CCT, which is also known as TRiC (TCP-1 ring complex), is a protein complex that assists in the folding of many essential cellular proteins [1,2]. Previous interactome studies have shown that CCT may be responsible for the proper folding of up to 10% of all cellular proteins [1]. Structurally, CCT is made of two ring structures positioned on top of each other. Each ring is comprised of eight paralogous subunits [3,4,5,6]. The rings form a folding chamber in the middle of the complex, which is large enough to enclose a 70 kDa protein [7]. Unfolded proteins bind in the chamber and their folding is completed in an ATP-dependent manner [4,6,8]. Detailed mechanistic studies of these folding events have shown that CCT employs a co-chaperone-mediated process to fold a subset of protein substrates [9,10]. One notable example is the phosducin-like protein 1 (PhLP1)-assisted folding of the G protein beta subunit (Gβ) by CCT. In its folding chamber, CCT binds the newly synthesized Gβ polypeptide, which is in a near-native state. Then, PhLP1 binds the CCT-Gβ complex, stabilizes the mature fold of Gβ, and triggers its release from CCT in a fully folded form [11,12]. The PhLP1-Gβ complex then associates with Gγ to form a stable Gβγ dimer that is ready to associate with Gα, which displaces PhLP1 and forms the complete G protein heterotrimer [11]. Currently, it is unknown if other CCT substrates require the assistance of PhLP1 for their proper folding.

The Gβ subunit has a 7-bladed β-propeller structure that contains seven WD40 repeats in its sequence [13,14]. Each repeat consists of about 40 amino acids, with the amino acid residues tryptophan and aspartate at the end, hence the name WD40. Indeed, it has been reported that the WD40 motif is the fourth most abundant motif observed in human proteins [15,16]. While WD40 proteins have five to seven repeats of the WD40 sequence motif that forms β-propeller structures with 7 blades, there is a great diversity among them with respect to their sequence and function, and they have been linked to a large group of diseases [17,18]. Protein interactome and immunoprecipitation studies have shown that some WD40 proteins with a β-propeller structure can associate with CCT [19,20]; however, only Gβ has been shown to recruit PhLP1 as a co-chaperone to assist in its folding.

The PhLP1 protein is found in almost all mammalian cell-types [21,22,23]. This broad expression pattern suggests that the function of PhLP1 is connected to a process applicable to most, if not all, cells and tissues. Members of the PhLP family have been shown to assist in the folding of actin and tubulin, and to regulate the cell cycle progression in yeast [21,24]. In mammalian cells, the main role of PhLP1 is to assist in the proper folding of the Gβ subunit and the formation of the Gβγ subunit dimer [11,12]. Since there are many other WD40 proteins with 7-bladed β-propeller structures like Gβ, it is likely that some of them would also bind and perhaps be folded by CCT with PhLP1 acting as a co-chaperone. In this study, proteins with a 7-bladed β-propeller structure were identified using biosequence analysis. The coding sequences of selected proteins were then subcloned and consequently transiently expressed in mammalian cells. Their binding to CCT and PhLP1 was determined by in vitro immunoprecipitations and immunoblotting. The obtained results reveal new binding partners for CCT and PhLP and that PhLP1 may have a much broader role as a co-chaperone for the folding of other WD40 proteins with a 7-bladed β-propeller structure.

## 2. Materials and Methods

### 2.1. Phylogenetic Analysis

**Figure 1:**
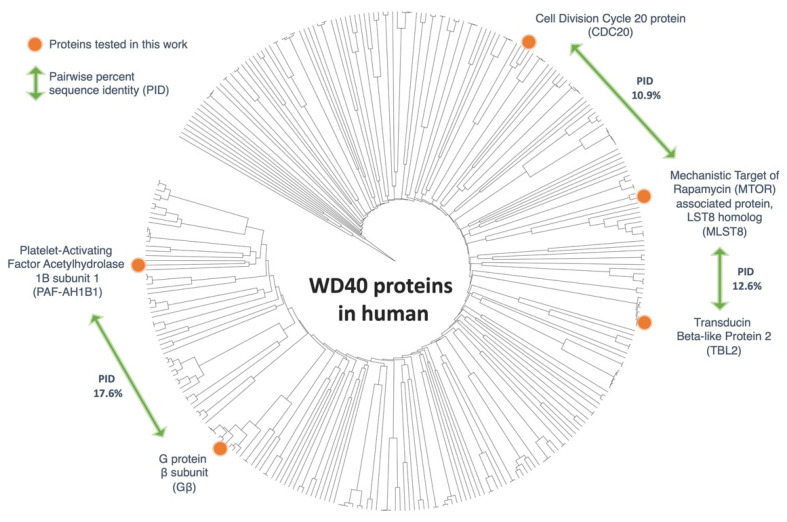
Phylogenetic analysis of WD40-motif-containing human proteins. A phylogenetic tree representing 318 WD40 sequences, identified using the bioinformatics filters described in the text. The protein sequences tested in this study are marked on this tree with orange circles. The percent sequence identity shown illustrates the diversity of the sequences used in this study.

To analyze the sequence relationship between WD-motif-containing proteins, a biosequence search was performed using HMMER (EMBL-EBI, Wellcome Genome Campus, Hinxton, Cambridgeshire, UK)) [25] against the UniProt database [26] using default settings. A series of filters were used to extract a representative set of sequences for visualization on a phylogenetic tree shown in Figure 1. Briefly, a pHMMER search was performed using the protein sequence of human Gβ, with search restriction set to include only Homosapien sequences. The full sequence FASTA was obtained, and duplicate sequences were removed using the CD-HIT suite [27]. Sequences that were likely to be incomplete (<200 amino acids) or multidomain proteins (>2-fold longer than human Gβ) were manually removed using the software Geneious (Biomatters, Ltd., Auckland, New Zealand) [28]. The final set of 318 sequences were aligned using MUSCLE alignment [29]. The all-to-all sequence identity and phylogenetic tree were calculated using Geneious with default options (UPGMA algorithm). The resulting Newick tree file was visualized using the online software iTOL (EMBL, Heidelberg, Germany) [30,31]. From the remaining sequences, four proteins with 7-bladed β-propeller structure and low average all-to-all pairwise sequence identity were selected for subcloning and consequent interaction studies. The four selected proteins were the Platelet-Activating Factor Acetylhydrolase 1B regulatory subunit 1 (PAF-AH1B1), Transducin Beta-Like Protein 2 (TBL2), Mechanistic Target of Rapamycin (MTOR) associated protein, LST8 homolog (MLST8), and Cell Division Cycle 20 protein (CDC20).

### 2.2. Preparation of Expression Constructs

DNA constructs containing the complete open reading frames (ORFs) of the selected four genes (TBL2, MLST8, CDC20, and PAF-AH1B1) were purchased from Open Biosystems (Huntsville, AL, USA). Each coding sequence was amplified by PCR with gene specific primers, and then inserted into the pcDNA™-DEST40 expression vector (ThermoFisher Scientific/Life Technologies, Waltham, MA, USA) using Gateway™ (ThermoFisher Scientific) recombination techniques. The stop codons were removed from the open reading frames to allow C-terminal fusion of the expressed proteins with the V5 tag provided by the vector. The sequence integrity of each of the four genes was confirmed by sequencing (GenScript, Piscataway, NJ, USA). The preparation of the c-Myc-tagged PhLP1 and the Flag-tagged Gβ expression constructs has been described in previously published work [11,32].

### 2.3. Cell Culture

Chinese hamster ovary cells (CHO-K1) (American Type Culture Collection (ATCC), Manassas, VA, USA) were cultured in complete growth media (DMEM/Ham’s F-12 50/50 with L-glutamine and 15 mM HEPES media (Corning, Corning, NY, USA), supplemented with 10% by volume fetal bovine serum (ThermoFisher)) under standard conditions (37 °C, in a humidified, 5% CO_2_ incubator, NuAire, Plymouth, MN, USA). Every three days, the cells were subcultured to maintain active growth. Cells beyond 20 passages were not used.

### 2.4. Transient Transfections

Cultured CHO-K1 cells were transfected with 2 µg of plasmid DNA (3 µg total, when two constructs were co-transfected) using the Lipofectamine 2000 reagent according to the manufacturer’s protocol (Invitrogen/ThermoFisher Scientific). The cells were harvested for subsequent applications 48 h after transfection.

### 2.5. Immunoprecipitations (IP) and Western Immunoblotting

Transfected CHO-K1 cells, in 6-well plates, were washed with phosphate-buffered saline (PBS) (ThermoFisher) and solubilized in 200 µL of immunoprecipitation (IP) buffer (PBS, pH 7.4, supplemented with 2% IGEPAL (Sigma-Aldrich, St. Louis, MO, USA), and Protease Inhibitor Cocktail for use with mammalian cell and tissue extracts (Sigma)), which was added right before the lysing of the cells. The lysates were passed 13 times through a 25 G needle and centrifuged at maximum speed for 8 min at 4 °C in a microfuge. The clarified lysates were incubated either with 2 µg of anti-V5 (ThermoFisher) or 2.5 µg of anti-CCTε (Bio-Rad/AbD Serotec, Hercules, CA, USA) monoclonal antibodies for 30 min., followed by incubation for additional 60 min. with 25 µL of a 50% slurry of Protein A/G Plus agarose (Santa Cruz Biotechnology, Dallas, TX, USA). After the incubations, the beads were washed three times with 400 µL of IP buffer. The precipitate was solubilized in 25 µL of 1.2× SDS-PAGE sample buffer (Bio-Rad), incubated in 90 °C water bath for 8 min, and 12 µL of the reaction products were resolved on 10% Tris-HCl Rgels (Bio-Rad). The resolved proteins were transferred from the gels to nitrocellulose paper and immunoblotted using anti-V5, anti-c-Myc (Sigma-Aldrich), anti-Flag (Sigma-Aldrich), or anti-CCTε antibodies. Immunoblots were developed with the ECL Plus chemiluminescence reagent (GE Healthcare/Amersham, Marlborough, MA, USA), detected with an ImageQuant LAS 4000 imager (GE Healthcare), and the band intensities were quantified using the ImageQuant TL software (GE Healthcare).

## 3. Results

### 3.1. Phylogenetic Analysis of WD40 Proteins

Proteins with WD40 repeats form one of the largest protein superfamilies. WD40 is also considered to be one of the top interacting motifs within the eukaryotic genome [16]. A biosequence search, using profile hidden Markov Models [25] against the UniProt database, showed 172,260 protein sequences similar to that of the human Gβ. To narrow down the number of sequences, the search was restricted to include only human sequences. This focused the study to 575 potential sequences. Then, sequence filters were applied, which discarded duplicate proteins, incomplete open reading frames (<200 amino acids), and sequences that are significantly longer than Gβ (>2-fold longer than Gβ). The modified search yielded a final set of 318 sequences with an average sequence identity of 14.4% (Figure 1).

From these sequences, four proteins were chosen based on their structural similarities, sequence diversity, and commercial availability of their complete open reading frames. One of the main objectives of this investigation was to determine if the Gβ-like structure, rather than the Gβ-like sequence, governs the interaction of the 7-bladed β-propeller WD40 proteins with CCT and PhLP1. For this reason, the selected proteins had a low average all-to-all pairwise sequence identity, but with a known or predicted 7-bladed β-propeller. The four proteins included in the analysis were the Platelet-Activating Factor Acetylhydrolase 1B regulatory subunit 1 (PAF-AH1B1), Transducin Beta-Like Protein 2 (TBL2), Mechanistic Target of Rapamycin (MTOR) associated protein, LST8 homolog (MLST8), and Cell Division Cycle 20 protein (CDC20). CDC20 is a known binding partner of CCT [33,34], but its binding to PhLP1 has not been studied. Multiple reports of yeast and human interactome studies, have shown that MLST8 interacts with CCT [2,19,35,36]. Cuellar at al. also revealed the cryo-EM structure of the human MLST8-CCT complex [37]. But, similarly to CDC20, the binding of MLST8 to PhLP1 has not been studied. TBL2 and PAF-AH1B1 have not been shown to interact with CCT and/or PhLP1.

### 3.2. Binding of 7-Bladed β-Propeller WD40 Proteins to CCT

To test for interactions with CCT, CDC20, MLST8, TBL2 and PAF-AH1B1 were transiently expressed in CHO-K1 cells and their binding to CCT was determined by CCT immunoprecipitation and western immunoblotting. To achieve that, constructs expressing each of the four proteins, tagged with the V5 tag, were prepared by insertion of their corresponding open reading frames (ORF) into the expression vector pcDNA3.1 DEST-40. The resulting constructs were then transiently expressed in CHO-K1 cells. The CHO-K1 cells were chosen because they naturally express CCT. The endogenous CCT was immunoprecipitated from the cell lysates using anti-CCTε antibodies, and the binding of the V5 tagged WD40 proteins was evaluated using immunoblotting against the V5 tag.

**Figure 2:**
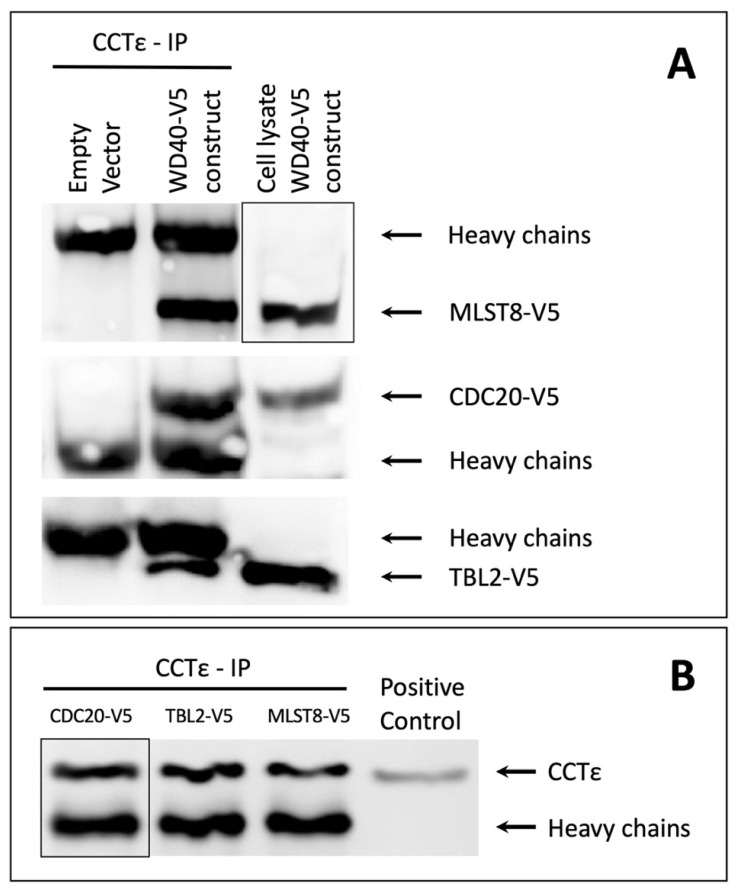
Immunoprecipitation of CCT. CHO-K1 cells transfected with one of the WD40-V5 expressing constructs (TBL2-V5, MLST8-V5, or CDC20-V5) or with an empty vector as a control, were lysed and treated with anti-CCTε antibodies and protein A/G beads. The immunoprecipitation products and lysates from WD40-V5 transfected cells, serving as positive controls, were analyzed using immunoblotting with anti-V5 antibodies to identify the V5 tagged WD40 proteins (**A**) or with anti-CCTε antibodies to identify CCT (**B**). Each panel shows a representative image of three repeats. The positive control for the CCT blot was a lysate from non-transfected cell.

The results reveal that three (CDC20, MLST8 and TBL2) of the four selected WD40 proteins bound to CCT (Figure 2, original immunoblots can be found in the Supplementary files).

The immunoblotting analyses of the CCT immunoprecipitations show clearly that CCT associates with each of the three proteins (Figure 2A). Cell lysates from transfected cells were used as positive controls for the individual proteins. The empty vector control samples confirm that non-specific proteins with a mass similar to that of the tested WD40-V5 proteins were not bound and precipitated by the anti-CCTε antibodies or the A/G beads. Panel B of Figure 2 shows that similar amounts of CCT were immunoprecipitated.

The interaction between CCT and CDC20 or MLST8 was expected and confirms previously published reports. The observed interaction between TBL2 and CCT identifies TBL2 as a novel binding partner of CCT. This observation strongly suggests that TBL2 associates with CCT in order to complete its folding and achieve a fully functional confirmation, similarly to Gβ, CDC20 and MSLT8. Unfortunately, our studies with PAF-AH1B1 did not produce reliable results (data not shown). On the immunoblots, PAF-AH1B1 did not appear as a well-defined band, which made the detection unreliable, and ultimately it was decided to exclude this protein from the study. Overall, the results show that all the selected Gβ-like WD40 proteins associate with CCT, except PAF-AH1B1, for which the results were inconclusive.

### 3.3. Binding of 7-Bladed β-Propeller WD40 Proteins to PhLP1

The results of Figure 2 suggest that diverse 7-bladed β-propeller WD40 proteins are folding substrates of CCT. Whether or not PhLP1 acts as a co-chaperone during the folding of these proteins is unknown. To examine this possibility and assess the binding of TBL2, MLST8, and CDC20 to PhLP1, additional co-immunoprecipitation experiments were carried out. To perform the immunoprecipitations, CHO-K1 cells were co-transfected with two plasmids, one expressing the c-Myc tagged PhLP1 and the other expressing one of the WD40 proteins tagged with V5. The cells were then lysed, and PhLP1 was immunoprecipitated with anti-Myc antibodies and Protein A/G beads. Subsequently, the binding of the WD40 proteins to the immunoprecipitated PhLP1 was analyzed using immunoblotting. As a positive control, we used Flag-tagged Gβ, which is a well-documented CCT and PhLP1 binder [11,12,38].

**Figure 3:**
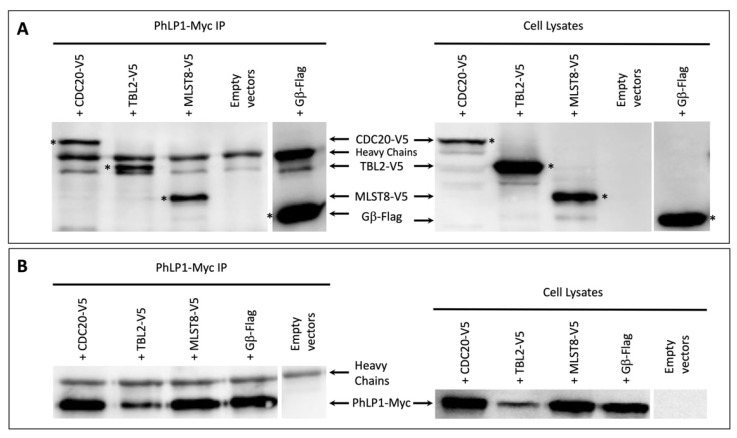
Immunoprecipitation of PhLP1-Myc. CHO-K1 cells, co-transfected with the PhLP1-Myc and one of the WD40-V5 expression constructs (TBL2-V5, MLST8-V5, or CDC20-V5) or with the Flag-tagged Gβ expression construct, were lysed. The lysates were then treated with anti-Myc antibodies and protein A/G agarose beads. The precipitated products and cell lysates were analyzed using western immunoblotting with the following antibodies: anti-V5 for detection of the V5-tagged WD40 proteins or anti-Flag for detection of Gβ (**A**); or anti-Myc for detecting PhLP1-Myc (**B**). All images are representative of at least three repeats, and * designates the band which corresponds to the protein name.

The results from the western immunoblotting show that all three proteins co-immunoprecipitated with PhLP1 (Figure 3, original immunoblots can be found in the Supplementary files).

Clear bands corresponding to CDC20, TBL2, and MLST8 were observed in the PhLP1 immunoprecipitates in addition to the antibody heavy chains (Figure 3A). The band observed below the Heavy Chains in the Empty Vectors but also in all samples, is most likely a degradation product of the heavy chains or a protein bound non-specifically to the anti-Myc antibody or the Protein A/G Plus agarose.

Interestingly, the PhLP1-Myc protein levels in the TBL2-V5 and PhLP1-Myc co-transfected cells (Figure 2B) appeared significantly lower compared to the other three samples. This was observed consistently, and it was most likely due to changes in the expression or half-life of PhLP1-Myc in the presence of TBL2-V5 and not as a result of an experimental error. Despite the low amounts of PhLP1-Myc, the results clearly show that TBL2-V5 interacts with PhLP1-Myc.

The binding of these β-propeller proteins to both PhLP1 and CCT indicates that they are likely to form a ternary complex with PhLP1 and CCT as has been observed with Gβ [12]. These observations strongly suggest that diverse subfamilies of WD40 proteins with 7-bladed β-propellers are folded by CCT, possibly with the assistance of PhLP1.

## 4. Discussion

The main objective of this work was to determine if diverse WD40 proteins that share a 7-bladed β-propeller structure associate with CCT and PhLP1. The binding of TBL2, MLST8 and CDC20, which are from different WD40 subfamilies (Figure 1), to CCT and PhLP1 indicates that this is the case and suggests a general mechanism of WD40 protein folding. CCT appears to have a broad role in β-propeller protein folding, while the contribution of PhLP1 seems more specific. For example, PhLP1 promotes the release of Gβ_1_ from CCT and the formation of the Gβγ dimer [11,12]. In contrast, PhLP1 enhances the binding of Gβ_5_ to CCT to promote Gβ_5_ folding [39], and PhLP1 did not appear to play an active role in MLST8 folding despite the fact that it binds both CCT and MLST8 [37].

The novel protein interactions between CCT and TBL2 or between PhLP1 and TBL2, MLST8, or CDC 20, identified in this report present valuable information and open numerous possibilities for future research aimed at understanding the folding of the β-propeller structure. While CCT facilitates the folding of its binding partners, the role of PhLP1 in the folding of Gβ-like proteins, such as CDC20 and TBL2, has yet to be established. Does PhLP1 assist in the folding of TBL2 and CDC20, or do these proteins engage other co-chaperones, or perhaps no co-chaperons at all? If PhLP1 does not assist in the folding of these and other proteins, then what role does it have in their function?

Structural analyses of the CCT-Gβ and CCT-MLST8 complexes revealed that even though Gβ and MLST8 have similar structures, they associate with CCT differently and their folding follows different mechanisms [12,37]. While Gβ associates with the apical domains of CCT, MLST8 associates deep within the CCT structure. The folding of both proteins is ATP-dependent, but ATP hydrolysis is sufficient for the releases only of the folded MLST8 and not for the release of Gβ. PhLP1 is required for the release of Gβ from CCT and for the subsequent formation of the Gβγ complex, but it has no role in the release of MLST8 from CCT or the formation of the MTORC1 complex. Growing evidence suggests that β-propellers achieve their mature confirmation in the folding chamber of CCT. Therefore, proteins with a known or predicted β-propeller structure are likely to bind and be folded by CCT. However, specific structural and sequence characteristics within the individual β-propellers would most likely determine and direct the location and type of interactions between CCT and β-propellers, and ultimately their folding. Revealing the cryo-EM structures and binding sites between CCT and CDC20, TBL2 or other 7-bladed β-propeller WD40 proteins would allow us to compare these interactions with those observed in the CCT-Gβ and CCT-MSLT8 complexes, which in turn would help us identify common and distinctive factors that govern the folding of this large protein family.

## 5. Conclusions

TBL2, CDC20 and MLST8, all WD40-motif proteins with 7-bladed β-propeller structure, were identified as novel binding partners for PhLP1. TBL2 was also revealed as a novel binding partner for CCT. PhLP1 and CCT bound all three proteins, suggesting that they may collaborate in the folding of this protein family. The identification of these novel binding partners for PhLP1 and CCT opens new possibilities for investigating the mechanism of folding of β-propeller WD40 proteins, and the role PhLP1 has in their function.

## Supplementary Materials

The following are available online at https://www.mdpi.com/article/10.3390/proteomes9040040/s1, Figure S1: Original immunoblots for Figure 2A, Figure S2: Original immunoblot for Figure 2B, Figure S3: Original immunoblots for Figure 3.
