# Supplementary material for: Novel Binding Partners for CCT and PhLP1 Suggest a Common Folding Mechanism for WD40 Proteins with a 7-Bladed Beta-Propeller Structure
Source: Proteomes. 2021 Oct 2;9(4):40. doi: 10.3390/proteomes9040040 (PMC8544692; doi:10.3390/proteomes9040040)

Figure S1: Original immunoblots for Figure 2A. The images shown on this figure are the original immunoblots used to generate Figure 2A. They show the co-immunoprecipitation of TBL2-V5 (A), CDC20-V5 (B) and MLST8-V5 (C) with CCT. A section of the blot shown in panel C (bracketed) was flipped horizontally in order to align the Empty Vector and WD40-construct samples in all three images for more clear representation.

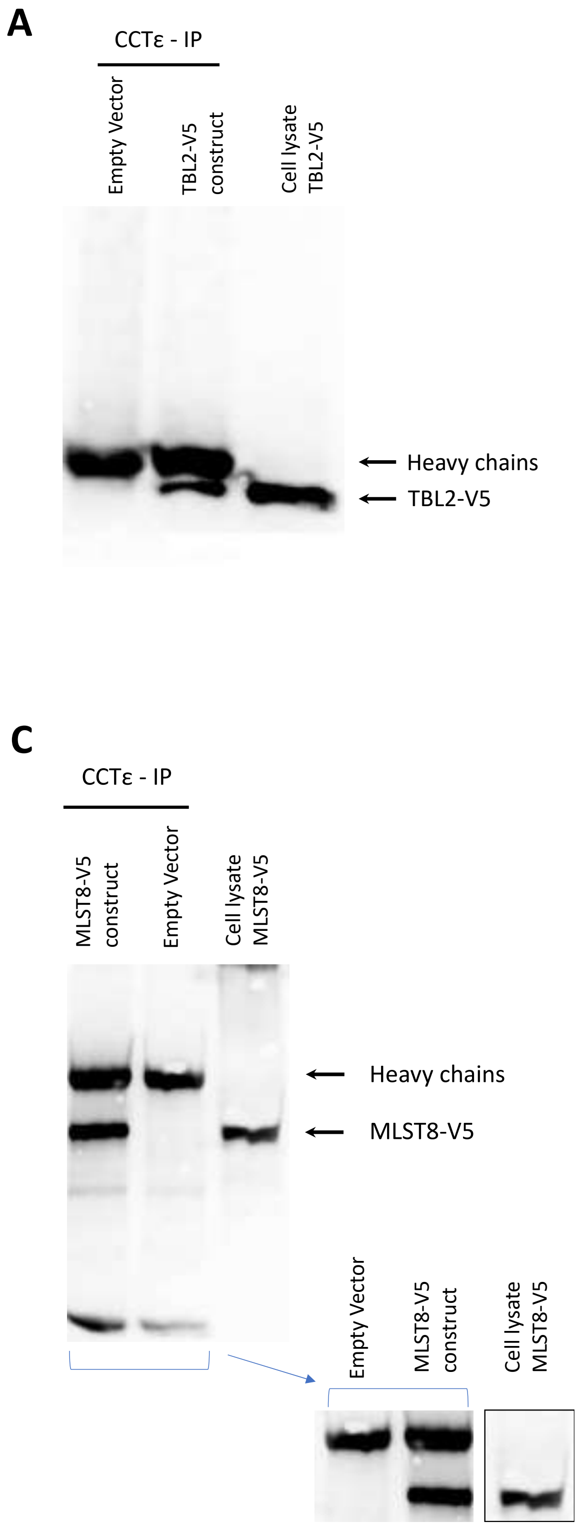

Figure S2. Original immunoblot for Figure 2B. Panel A shows the original immunoblot used to generate Figure 2B. PAF-AH1B1-V5, shown in red on the image, was removed from this study because of detection difficulties and inconclusive results. During the preparation of the final version of Figure 2B, the PAF-AH1B1-V5 section of the blot was excised and the CDC20-V5 section was shifted sideways and positioned next to the TBL2-V5 lane. The image shown in panel B was not used in Figure 2. However, because the blots used in Figure 2 did not have a protein molecular weight standard, we included this blot, which contains all studied WD40 proteins and a molecular weight standard. This specific blot represents the same experiment described in Figure 2, but the immunoblot was developed using a colorimetric method with 4-chloronaphtol.

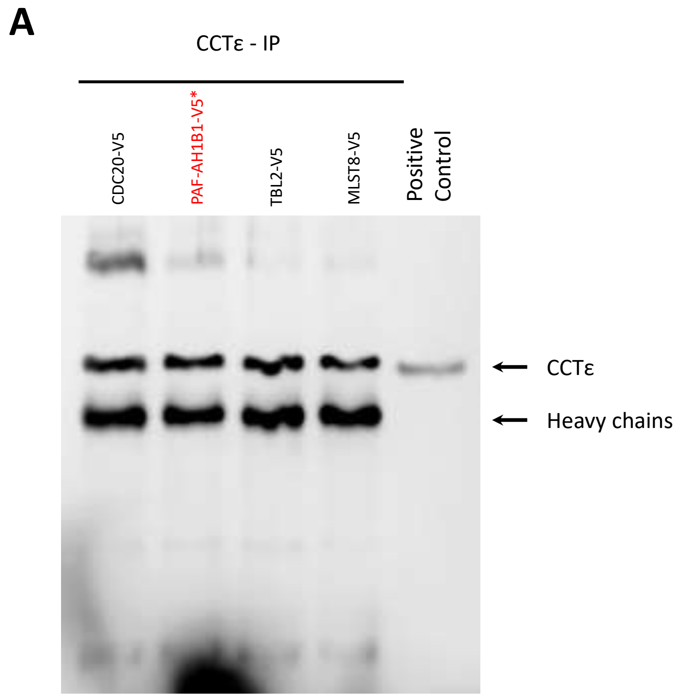

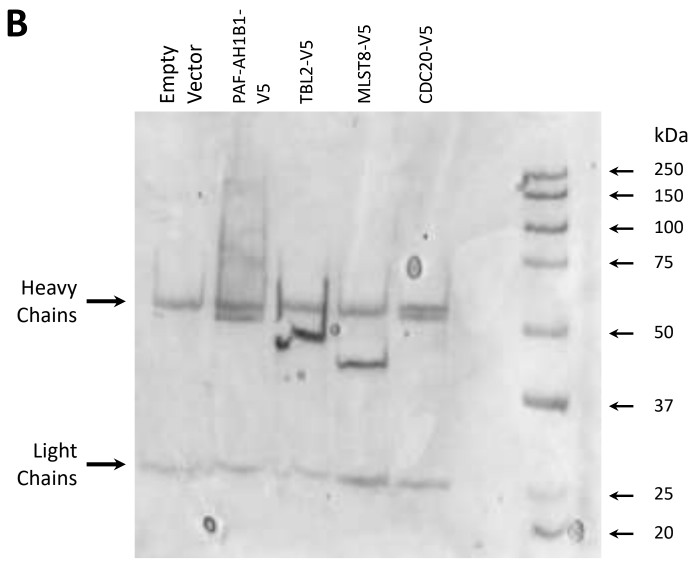

Figure S3. Original immunoblots for Figure 3. Panels A and B show the original immunoblot used to generate Figure 3A. Panel A shows the WD40-V5 proteins which co-immunoprecipitated with PhLP1 and their expression in the transfected cells. Panel B shows Flag-Gβ expression and co-immunoprecipitation with PhLP1. The immunoblots shown on panel C are the original blots used to generate Figure 3B. They show the expression of PhLP1 in transfected cells, and immunoprecipitated PhLP1 with anti-Myc antibodies.

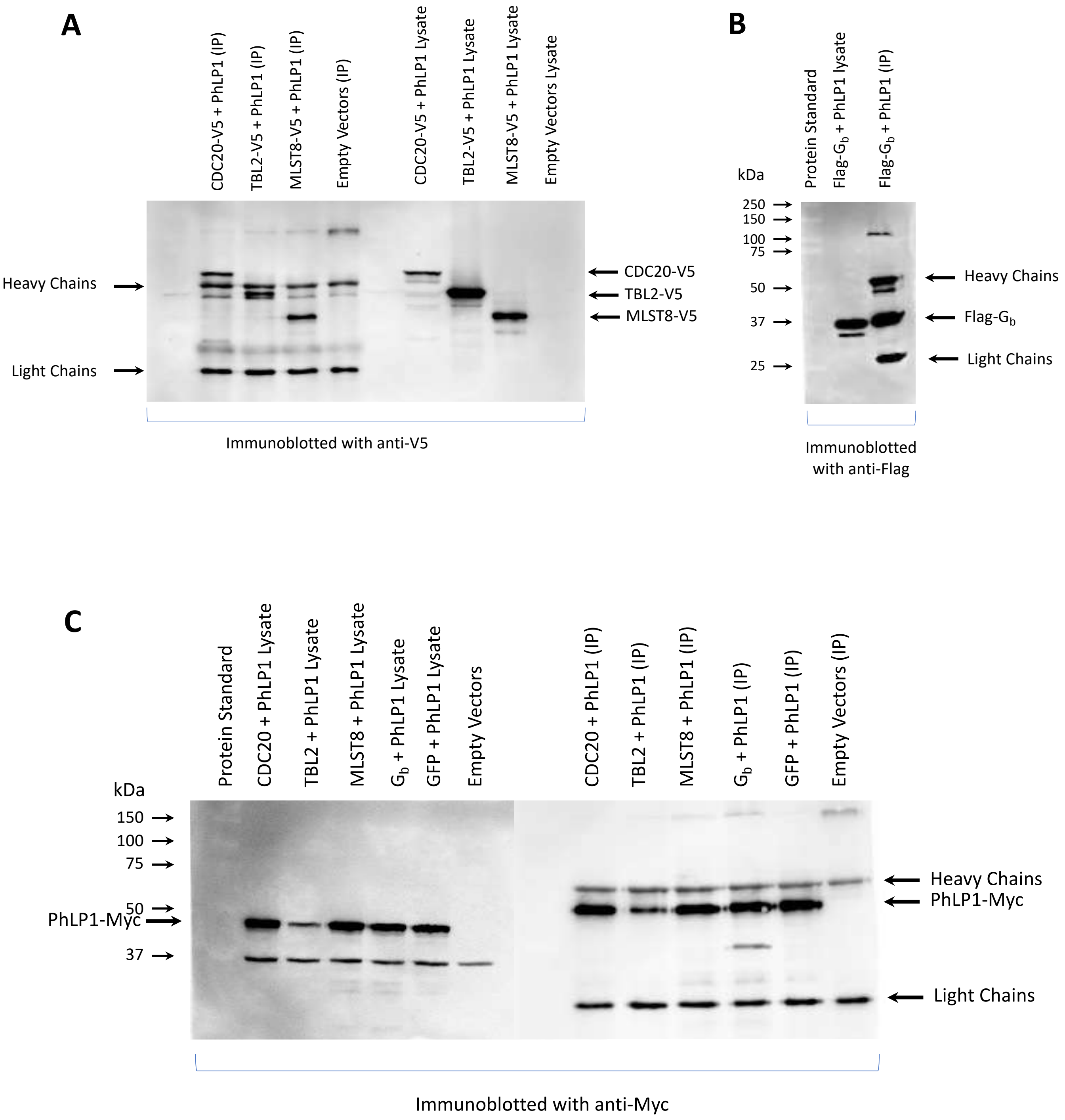

Supplement: Supplementary file 1 [file proteomes-09-00040-s001.zip › proteomes-1344710-suppl.pdf]
